# Supplementary material for: miR-30c-5p Gain and Loss of Function Modulate Sciatic Nerve Injury-Induced Nucleolar Stress Response in Dorsal Root Ganglia Neurons
Source: Int J Mol Sci. 2024 Oct 24;25(21):11427. doi: 10.3390/ijms252111427 (PMC11547303; doi:10.3390/ijms252111427)
Supplement: Supplementary file 1 [file ijms-25-11427-s001.zip › ijms-3238743-supplementary.pdf]

Descriptive statistics and effect size for results shown in figure 1

Figure 1I: Percentage of damaged neurons

Source	*SS*	*df*	*F (DFn, DFd)*	*p*	η^2
Nerve injury	2348	1	F (1, 8) = 45.84	p = 0.001	**0.97**
miR-30c-5p mimic	430	1	F (1, 8) = 296.5	p < 0.001	**0.87**
Interaction	363	1	F (1, 8) = 54.30	p < 0.001	**0.85**
Residual	63.35	8			

Condition	**Mean ± SD**	**n**
Sham + Vehicle	2.56 ± 1.76	3
Sham + miR-30c-5p mimic	3.53 ± 0.60	3
SNI Day 5	19.53 ± 1.49	3
SNI + miR-30c-5p mimic	42.51 ± 5.10	3

Figure 1J: Percentage of damaged neurons

Source	*SS*	*df*	*F (DFn, DFd)*	*p*	η^2
Nerve injury	672.7	1	F (1, 8) = 23.83	p = 0.0012	**0.93**
miR-30c-5p inhibitor	123.2	1	F (1, 8) = 123.5	p < 0.001	**0.73**
Interaction	129.8	1	F (1, 8) = 22.61	p = 0.0014	**0.74**
Residual	43.57	8			

Condition	**Mean ± SD**	**n**
Sham + Vehicle	2.56 ± 1.76	3
Sham + miR-30c-5p inhibitor	2.73 ± 0.78	3
SNI Day 10	24.11 ± 3.12	3
SNI + miR-30c-5p inhibitor	11.13 ± 2.89	3

Figure 1K: Percentage of cells with eccentric nucleus

Source	*SS*	*df*	*F (DFn. DFd)*	*p*	η^2
Nerve injury	155.8	1	F (1, 8) = 6.071	p = 0.0391	**0.57**
miR-30c-5p mimic	89.33	1	F (1, 8) = 10.89	p = 0.0109	**0.43**
Interaction	86.83	1	F (1, 8) = 6.245	p = 0.0370	**0.43**
Residual	114.4	8			

Condition	**Mean ± SD**	**n**
Sham + Vehicle	0.24 ± 0.28	3
Sham + miR-30c-5p mimic	0.57 ± 0.14	3
SNI Day 5	2.27 ± 1.21	3
SNI + miR-30c-5p mimic	13.11 ± 7.45	3

Figure 1L: Percentage of cells with eccentric nucleus

Source	*SS*	*df*	*F (DFn. DFd)*	*p*	η^2
Nerve injury	2.70	1	F (1, 8) = 14.21	p = 0.0055	**0.76**
miR-30c-5p inhibitor	1.268	1	F (1, 8) = 26.68	p = 0.0009	**0.60**
Interaction	1.44	1	F (1, 8) = 12.49	p = 0.0077	**0.63**
Residual	0.81	8			

Condition	**Mean ± SD**	**n**
Sham + Vehicle	0.24 ± 0.28	3
Sham + miR-30c-5p inhibitor	0.47 ± 0.13	3
SNI Day 10	2.09 ± 0.40	3
SNI + miR-30c-5p inhibitor	1.19 ± 0.84	3

Descriptive statistics and effect size for results shown in figure 3

Figure 3I: Percentage of neurons with segregated nucleolus

Source	*SS*	*df*	*F (DFn. DFd)*	*p*	η^2
Nerve injury	2770	1	F (1, 8) = 56.39	p<0.0001	**0.96**
miR-30c-5p mimic	552.8	1	F (1, 8) = 194.5	p<0.0001	**0.82**
Interaction	803.1	1	F (1, 8) = 38.82	p=0.0003	**0.87**
Residual	113.9	8			

Condition	**Mean ± SD**	**n**
Sham + Vehicle	6.67 ± 1.95	3
Sham + miR-30c-5p mimic	3.89 ± 1. 67	3
SNI Day 5	20.70 ± 6.91	3
SNI + miR-30c mimic	50.64 ± 1.59	3

Figure 3J: Percentage of neurons with segregated nucleolus

Source	*SS*	*df*	*F (DFn. DFd)*	*p*	η^2
Nerve injury	427.9	1	F (1, 8) = 26.68	p=0.0009	**0.87**
miR-30c-5p inhibitor	336.9	1	F (1, 8) = 56.47	p<0.0001	**0.84**
Interaction	202.2	1	F (1, 8) = 44.45	p=0.0002	**0.76**
Residual	60.63	8			

Condition	**Mean ± SD**	**n**
Sham + Vehicle	6.67 ± 1.95	3
Sham + miR-30c inhibitor	4.29 ± 0.63	3
SNI Day 10	26.83 ± 2.12	3
SNI + miR-30c inhibitor	8.02 ± 1.97	3

F-values and variables analysed for each result shown in figure 5.

Percentage of neurons with 0, 1, 2 or more Cajal bodies	
**Figure 5E**	0 CBs: $F(3, 10) = 6.859$ $p < 0.05$
**Figure 5F**	0 CBs: $F(3, 11) = 185.6$ $p < 0.001$<br>≥2 CBs: $F(3, 10) = 3.48$ $p < 0.001$
